# Cold Water as an Antipyretic

**Published:** 1894-10

**Authors:** 


COLD WATER AS AN ANTIPYRETIC.—Dr. Fisher has arrived at the following conclusions:

1. That cold water is the best antipyretic used to-day.
2. That it is easily obtainable, and is therefore adapted to all classes of cases, both rich and poor.
3. The mode of application is so simple that it adapts itself to the hospital, and equally as well to private practice, and can be applied without any distinct training.
4. Cautiously given it stimulates.
5. Carelessly used and longer than required, it depresses, and will produce subnormal temperature.
6. That rectal temperature should be taken, and the bath at once discontinued when temperature falls to 101 degrees, as it will then continue to fall.
7. That a stimulant administered before the bath may be necessary, and should be given where there is a feeble heart.
8. That the temperature indicates when to commence, and when, also, to discontinue the hydropathic treatment.
9. Unnecessary blanketing after the bath is injurious, and will produce copious perspiration, which I believe weakens the patient.
10. The temperature of the room should always be between 68 and 72 degrees.—*Post Graduate.*

